# What, if anything, are hybrids: enduring truths and challenges associated with population structure and gene flow

**DOI:** 10.1111/eva.12380

**Published:** 2016-04-26

**Authors:** Zachariah Gompert, C. Alex Buerkle

**Affiliations:** ^1^ Department of BiologyUtah State UniversityLoganUT USA; ^2^ Department of BotanyUniversity of WyomingLaramieWY USA

**Keywords:** admixture, conservation biology, genetic ancestry, hybridization, population genetics

## Abstract

Hybridization is a potent evolutionary process that can affect the origin, maintenance, and loss of biodiversity. Because of its ecological and evolutionary consequences, an understanding of hybridization is important for basic and applied sciences, including conservation biology and agriculture. Herein, we review and discuss ideas that are relevant to the recognition of hybrids and hybridization. We supplement this discussion with simulations. The ideas we present have a long history, particularly in botany, and clarifying them should have practical consequences for managing hybridization and gene flow in plants. One of our primary goals is to illustrate what we can and cannot infer about hybrids and hybridization from molecular data; in other words, we ask when genetic analyses commonly used to study hybridization might mislead us about the history or nature of gene flow and selection. We focus on patterns of variation when hybridization is recent and populations are polymorphic, which are particularly informative for applied issues, such as contemporary hybridization following recent ecological change. We show that hybridization is not a singular process, but instead a collection of related processes with variable outcomes and consequences. Thus, it will often be inappropriate to generalize about the threats or benefits of hybridization from individual studies, and at minimum, it will be important to avoid categorical thinking about what hybridization and hybrids are. We recommend potential sampling and analytical approaches that should help us confront these complexities of hybridization.

## Introduction

Sexual reproduction that involves mating with other individuals (outcrossing rather than selfing) and meiotic recombination mix alleles among different genomic backgrounds. Physical dispersal of individuals before reproduction moves alleles farther from where they originated by mutation and is referred to as gene flow. At some point, crosses can occur between individuals that are unrelated enough that we refer to these as hybrids. Although hybridization has sometimes been viewed as an unimportant dead end, there is a long history of interest in hybridization as a potent creative and destructive evolutionary process (e.g. Stebbins 1950; Ellstrand 1992; Rieseberg and Wendel 1993; Buerkle et al. 2003; Arnold 2006). Numerous cases where hybridization and introgression have had substantial ecological or evolutionary consequences in plants are known. For example, hybridization between the sunflower species *Helinathus annuus* and *Helinathus petiolaris* resulted in multiple distinct hybrid species (Rieseberg et al. 1990, 1995, 2003a), and hybridization in *Populus* affects community composition and ecosystem processes (Driebe and Whitham 2000; Martinsen et al. 2000; Whitham et al. 2006; Floate et al. 2016). Hybridization is particularly common among oak species, where it may spread or generate adaptive genetic variation and where it has been proposed as a key component of natural and human‐induced invasions (Petit et al. 2004; Moran et al. 2012).

The consequences of hybridization are directly relevant to aspects of conservation biology and agriculture. Hybridization, whether natural or human induced, can affect the origin, maintenance, and loss of biodiversity (Rhymer and Simberloff 1996; Wolf et al. 2001; Buerkle et al. 2003; Zalapa et al. 2010; Muhlfeld et al. 2014). Hybridization in plants could help endemic species survive periods of climate change (Becker et al. 2013) or result in extinction, when, for example, native species are assimilated by non‐native species or experience demographic decline due to outbreeding depression (Ellstrand 1992; Levin et al. 1996; Balao et al. 2015; Gómez et al. 2015). Introgressive hybridization also occurs between crops and their wild relatives, and this too can have beneficial or detrimental consequences for biodiversity (Linder et al. 1998; Ellstrand et al. 2013; Hufford et al. 2013; Warschefsky et al. 2014). Of particular interest is the potential for crop–wild hybridization to allow modified or engineered genes to escape into the wild, which could negatively affect native species or increase public distrust of genetically modified crops (Ellstrand 2001; Stewart et al. 2003; Chapman and Burke 2006; Garnier et al. 2014). Another practical issue is whether and under what conditions hybrid populations or taxa warrant conservation efforts. Hybrids were not granted protection under the US Endangered Species Act, but this was questioned in a federal rule proposed in 1996 (this rule was never adopted; Allendorf et al. 2001, 2013). The proposed federal rule used the term ‘intercross’ rather than ‘hybrid’ to avoid a negative connotation of the latter (Allendorf et al. 2013) and we suspect that some people would view even natural hybrids as less worthy of protection than ‘pure’ species (e.g. the decision to conserve eastern wolves has in part been based on species or hybrid status; Rutledge et al. 2015). Clearly, the potential outcomes and practical consequences of hybridization are multifarious, and thus, different cases of hybridization will need to be treated differently.

Confronting this complexity requires careful consideration of what hybridization is, and when distinguishing among different processes is necessary and possible. The recognition of hybrids between named taxa is relatively uncontroversial, but it is somewhat poorly resolved as to what distance of a cross constitutes hybridization, and what therefore qualifies as a hybrid (Harrison 1993; Arnold 2006; Allendorf et al. 2013). Similarly, different histories of gene flow and selection, such as primary divergence versus secondary contact, have been referred to as hybridization (Barton and Hewitt 1985). However, discriminating among these different histories could be necessary from a management perspective, if, for example, we are to treat cases of natural and human‐induced hybridization differently as suggested by Allendorf et al. (2001). Unfortunately, different histories of hybridization can generate very similar or identical patterns of genetic and phenotypic variation (e.g. Barton and Hewitt 1985; Kruuk et al. 1999; Barton and de Cara 2009). This means we might not always be able to distinguish different histories even when doing so would be useful.

In this article, we review and discuss ideas that are relevant to recognition of hybrids and supplement these with simulations to illustrate important contrasts. We acknowledge that is atypical to have a paper contain review, synthesis of concepts and novel simulations, but we think the combination can be useful. The issues we address have a relatively long history, some of which is underappreciated, and clarifying these ideas should have practical consequences for managing hybridization and gene flow in plants. A reexamination of some of these points is worthwhile too because recent population genomic studies have led to a greater appreciation of variation within species and genomic heterogeneity in differentiation between species or populations (e.g. Martin and Orgogozo 2013; Gompert et al. 2014; Mandeville et al. 2015). Additionally, we have learned more about models and approaches that can be used to describe patterns of variation in hybrids (Patterson et al. 2012; Gompert and Buerkle 2013). Along these lines, it is important to recognize what we can and cannot infer about hybrids and hybridization from molecular data; in other words, we must be aware that genetic data provide incomplete information about hybridization. Our simulations and discussion focus on patterns of variation when hybridization is recent and populations are polymorphic; this contrasts with the bulk of theoretical work that concerns long‐term equilibrium outcomes of hybridization and often is most applicable when hybridizing taxa exhibit fixed differences. This distinction increases the novelty of our results and makes them particularly informative for applied issues and contemporary hybridization following recent ecological change. In the following, we first address the question of what constitutes hybridization and then turn to the definition of hybrids. We combine literature review and new simulations to answer these questions and conclude each section with recommendations for applied studies of hybridization and gene flow in plants.

## What, if anything, is hybridization

Hybridization has been variously defined as interbreeding between different species or subspecies, distinct populations or cultivars, or any individuals with heritable phenotypic differences (Stebbins 1950; Barton and Hewitt 1985; Harrison 1993; Allendorf et al. 2001; Arnold 2006). However, such distinctions downplay the continuous nature of genetic and phenotypic differentiation and distract from the fact that gene flow can have similar consequences anywhere along this continuum (Mayr 1963; Mallet et al. 2007; Martin and Orgogozo 2013). For example, because of population genetic structure and local adaptation within species, intraspecific gene flow can have positive, negative, or negligible effects on populations that are similar to those of interspecific gene flow (e.g. Ellstrand 1992; Kremer et al. 2012; Nosil et al. 2012; Roe et al. 2014). Moreover, the consequences of interspecific gene flow frequently depend on the specific individuals involved, because of polymorphisms within and among conspecific populations (Sweigart et al. 2007; Escobar et al. 2008; Good et al. 2008; Gompert et al. 2013). In other words, it is the evolutionary and ecological consequences of gene flow that should be considered when defining hybridization. Importantly, the consequences of gene flow do not depend on taxonomy or a specific definition of species, but rather on the nature of differences between groups. Of course, such differences also represent a continuum, and thus, an unambiguous and objective definition of hybridization as something distinct from gene flow is not likely possible. With that said, we think it is useful to reserve the term hybridization for cases where outcrossing and gene flow occur between populations that differ, at least quantitatively, at multiple heritable characters or genetic loci that affect fitness. Thus, we argue that the distinction between gene flow and hybridization is fuzzy and quantitative, rather than discrete and qualitative. While such a view could complicate management decisions, we think it more accurately captures patterns of variation in nature.

Different histories or geographies of gene flow and selection have often been referred to as hybridization. For example, several authors have argued that both primary divergence with gene flow and gene flow following secondary contact (i.e. gene flow after a prolonged period of geographic separation with very little or no gene flow) constitute hybridization (Barton and Hewitt 1985). We think that the case for secondary contact is uncontroversial, but that informed opinions might differ about whether primary divergence includes hybridization. Certainly, primary divergence is not the common conception of hybridization in conservation biology (Allendorf et al. 2001, 2013). Likewise, hybrid zones maintained primarily by exogenous (environment dependent) versus endogenous (environment‐independent) selection have been classified and treated similarly. However, management efforts could benefit from distinguishing among these different histories and processes. We might be more inclined to intervene when secondary contact occurs after an anthropogenic disturbance than when primary divergence occurs, even if the latter takes place in a disturbed area.

An equally important question is whether and under what conditions we can in fact discriminate among these different cases. On the one hand, theory shows that over the long‐term, primary divergence and secondary contact with exogenous or endogenous selection have similar equilibrium conditions and result in similar geographic patterns of genetic and phenotypic variation (Endler 1977; Barton and Hewitt 1985; Kruuk et al. 1999; Navarro and Barton 2003; Barton and de Vladar 2009; Barton 2013; Flaxman et al. 2014). However, it is also true that well‐documented examples of these different cases are known. For example, convergent clines in flowering time in sunflowers are best explained by primary divergence driven by exogenous selection (Blackman et al. 2011; Kawakami et al. 2011), whereas hybridization between *H. annuus* and *H. petiolaris*, which are not sister species, can be attributed to secondary contact (Rieseberg 1991). Additionally, the bulk of evidence suggests that many classic hybrid zones are tension zones maintained by endogenous selection (reviewed in Barton and Hewitt 1985). Consistent with this, Dobzhansky–Muller incompatibilities (DMIs) have been documented in several plant taxa, such as *Mimulus* and *Solanum* (Sweigart et al. 2007; Moyle and Nakazato 2010).

Here, we ask when genetic analyses commonly used to study hybridization might mislead us about the history or nature of gene flow and selection. We are particularly interested in cases where being misled could affect decisions in applied science. We consider primary divergence versus secondary contact, and neutral evolution versus selection on a quantitative trait along an environmental gradient or reduced hybrid fitness due to intrinsic epistatic incompatibilities (i.e. DMIs). We simulate genetic data under each of these conditions and then summarize the results by (i) examining allele frequency and trait clines, (ii) summarizing genetic variation with principal component analysis (PCA), and (iii) estimating admixture proportions. Our goal is not an exhaustive evaluation of these methods, but rather to provide illustrative examples of the potential to be misled by genetic data. We then turn to the related problem of finite sampling. In particular, we show that sparse population sampling when organisms are continuously distributed can lead to false inferences about population structure. That is to say, clinal variation can appear more demic and even suggestive of hybrid speciation. Importantly, and in contrast to most theoretical work on hybridization or hybrid zones, our simulations incorporate shared polymorphism across populations (or species), rather than focusing on genetic markers with fixed differences. This is realistic in general and better reflects the current generation of molecular data (e.g. SNPs identified and scored through genotyping‐by‐sequencing or exome sequencing).

### Simulations and analyses

We used individual‐based, genetically explicit simulations to generate pseudo‐data under different demographic and evolutionary histories. Simulations were conducted using the program nemo version 2.3.44 (Guillaume and Rougemont 2006). Generations were discrete, and each generation consisted of the following ordered events: breeding, dispersal, viability selection (some histories), and aging. Patches were arranged according to a 1‐D stepping‐stone model with dispersal allowed only between adjacent patches (dispersal off the outer‐edges of the patch vector was allowed). We assumed logistic growth within each patch with a carrying capacity of 5000 individuals and a mean fecundity of two. Genomes consisted of a single chromosome with a recombinational map length of one Morgan. We tracked 200 neutral bi‐allelic SNPs in all simulations, and 10 quantitative trait SNPs or DMI SNPs in relevant subsets of the simulations. In all cases, mutation rates were 0.0001 per locus per generation and SNPs were distributed according to a random uniform distribution along the recombinational map of the chromosome (this included neutral and non‐neutral SNPs). Simulations lasted 2000 generations.

Starting allele frequencies were generated for neutral markers, quantitative trait SNPs and DMI SNPs to mimic secondary contact or primary divergence (Figure S1). Ancestral allele frequencies were first generated for neutral SNPs by sampling from a beta distribution with *α* and *β* equal to 20 (this distribution has a mean of 0.5 and a standard deviation of 0.08). We then obtained initial allele frequencies for the two taxa experiencing secondary contact by sampling from $\text{beta}(\alpha =\pi \frac{1-F}{F}\;,\beta =\left(1-\pi \right)\frac{1-F}{F})$, where *π* is the ancestral allele frequency for the SNP and *F* corresponds to $F_{\mathrm{ST}}$ (Balding and Nichols 1995; Falush et al. 2003), which was set to 0.3 (i.e. substantial population genetic differentiation). We assigned one set of allele frequencies to patches 1–5 and a different set of allele frequencies to patches 6–10. We used the same procedure to generate initial neutral allele frequencies for primary divergence, except the same allele frequencies were assigned to all 10 patches. We initialized quantitative SNPs by assuming the two taxa were perfectly adapted to alternative ends of the patch vector (secondary contact; mean phenotypes of −0.5 and 0.5 were used for patches 1–5 and 6–10, respectively), or by setting the mean phenotype in each patch equal to 0 (primary divergence). We initialized DMI SNPs with different taxa fixed for different sets of derived alleles, such that no fitness reduction occurred within taxa but hybrids would experience reduced fitness (secondary contact), or with all populations fixed for the ancestral allele.

We then simulated five replicate data sets with the following conditions: neutral evolution following secondary contact (no DMIs and no effect of the quantitative trait on fitness), exogenous selection along an environmental gradient with primary divergence, exogenous selection along an environmental gradient following secondary contact, exogenous selection at a sharp ecotone with primary divergence, exogenous selection at a sharp ecotone following secondary contact, endogenous selection caused by DMIs with primary divergence, and endogenous selection caused by DMIs following secondary contact (summarized in Table 1). We repeated all simulations with migration rates of 0.01 and 0.001. Exogenous selection was based on a single quantitative trait that was under stabilizing selection in each patch; we used a Gaussian fitness function with mean *μ* and variance 0.5. *μ* varied from −0.5 to 0.5 in steps of 0.1 (most patches) or 0.2 (patches 5 and 6) between patches for the environmental gradient, and was set to −0.5 (patches 1–5) or 0.5 (patches 6–10) for the sharp ecotone. This means that an individual perfectly adapted to one end of the patch vector would have relative fitness of 0.37 at the other end. DMIs were modeled as negative fitness effects between derived alleles at pairs of SNPs. Considering a single locus pair, we assumed the double homozygote for different derived alleles had a fitness of 0.6, and an individual heterozygous at one locus and homozygous for derived alleles at the other had a fitness of 0.8; all other genotypes had a fitness of 1.0. We assumed fitness was absolute (not relative) and multiplicative across DMIs.

**Table 1:** Summary of conditions for simulations conducted with nemo (five replicates each)

Geography	Selection	Migration rate
Secondary contact	None	0.001
Primary divergence	Exogeneous, smooth gradient	0.001
Secondary contact	Exogeneous, smooth gradient	0.001
Primary divergence	Exogeneous, sharp ecotone	0.001
Secondary contact	Exogeneous, sharp ecotone	0.001
Primary divergence	Endogenous (DMIs)	0.001
Secondary contact	Endogenous (DMIs)	0.001
Secondary contact	None	0.01
Primary divergence	Exogeneous, smooth gradient	0.01
Secondary contact	Exogeneous, smooth gradient	0.01
Primary divergence	Exogeneous, sharp ecotone	0.01
Secondary contact	Exogeneous, sharp ecotone	0.01
Primary divergence	Endogenous (DMIs)	0.01
Secondary contact	Endogenous (DMIs)	0.01

DMI, Dobzhansky–Muller incompatibilities.

Additional data were simulated to evaluate the effect of limited sampling on inference. Our primary motivations were to determine whether sampling gaps would provide false evidence of discrete population clusters or a lack of hybrids when the underlying population structure was continuous (i.e. with isolation by distance). Here, we assumed neutral primary divergence in a 1‐D stepping‐stone model with 50 patches, each with a carrying capacity of 2500 individuals and a dispersal rate between neighboring patches of 0.001 (our focus on neutral primary divergence reflects our interest in isolation by distance). We initialized neutral allele frequencies as described above. We analyzed either samples from all 50 patches (50 or 5 individuals each), from sets of four patches at the edges and center of the patch vector (50 individuals each), and from the 12 center patches (50 individuals each).

We used three common analytical approaches to quantify and summarize patterns of genetic variation from the simulations: (i) character and allele frequency clines, (ii) ordination via PCA, and (iii) inference of admixture proportions. We plotted geographic clines in allele frequencies at all neutral SNPs and for the quantitative trait (with the exceptions of DMI simulations, which did not include a quantitative trait). Allele frequencies were polarized such that the rarer allele in the first patch was shown. We conducted PCA on the centered genotype data from 50 individuals from each patch in each simulation (i.e. 500 individuals total for most simulations) using the prcomp function in R (R Development Core Team 2015). Genotypes were coded as 0, 1, or 2 copies of one allele at each locus. We estimated admixture proportions using these same genetic data. We used the program admixture version 1.23 (Alexander et al. 2009) for this, which fits the same model as the admixture model in structure Pritchard et al. (2000), but uses maximum likelihood rather than Bayesian inference. We used the block‐relaxation method for parameter estimation with a tolerance of 0.0001 and the Quasi‐Newton algorithm for convergence acceleration.

**Figure 1:**
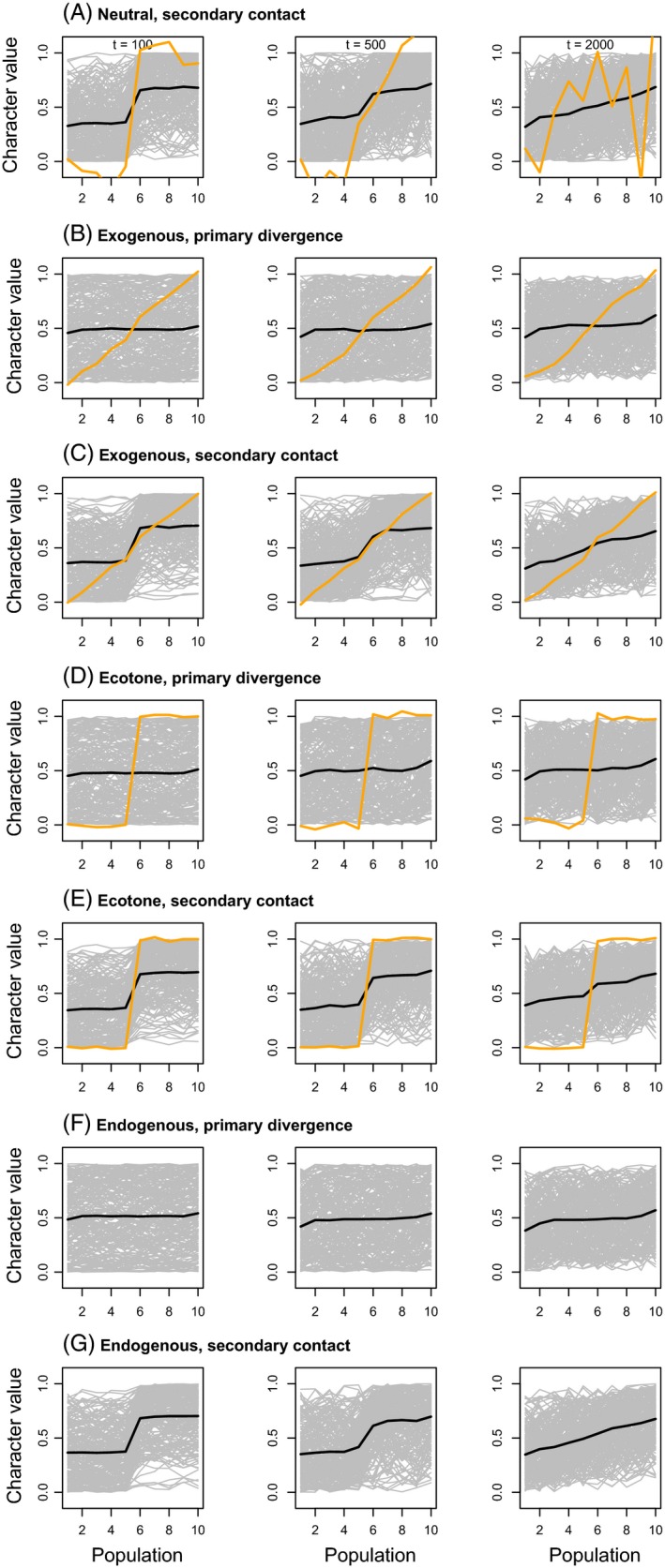
Plots show neutral allele frequency (gray) and quantitative trait (orange) clines from simulated data with a migration rate of 0.001. The mean allele frequency cline with SNPs polarized such that the allele plotted was rarer in patch 1 than patch 10 is depicted with a black line. Clines after 100, 500, and 2000 generations are shown. Results from a single simulation are shown, but replicate simulations produced qualitatively similar results. Clines from simulations with a higher migration rate of 0.01 are shown in Figure S2.

**Figure 2:**
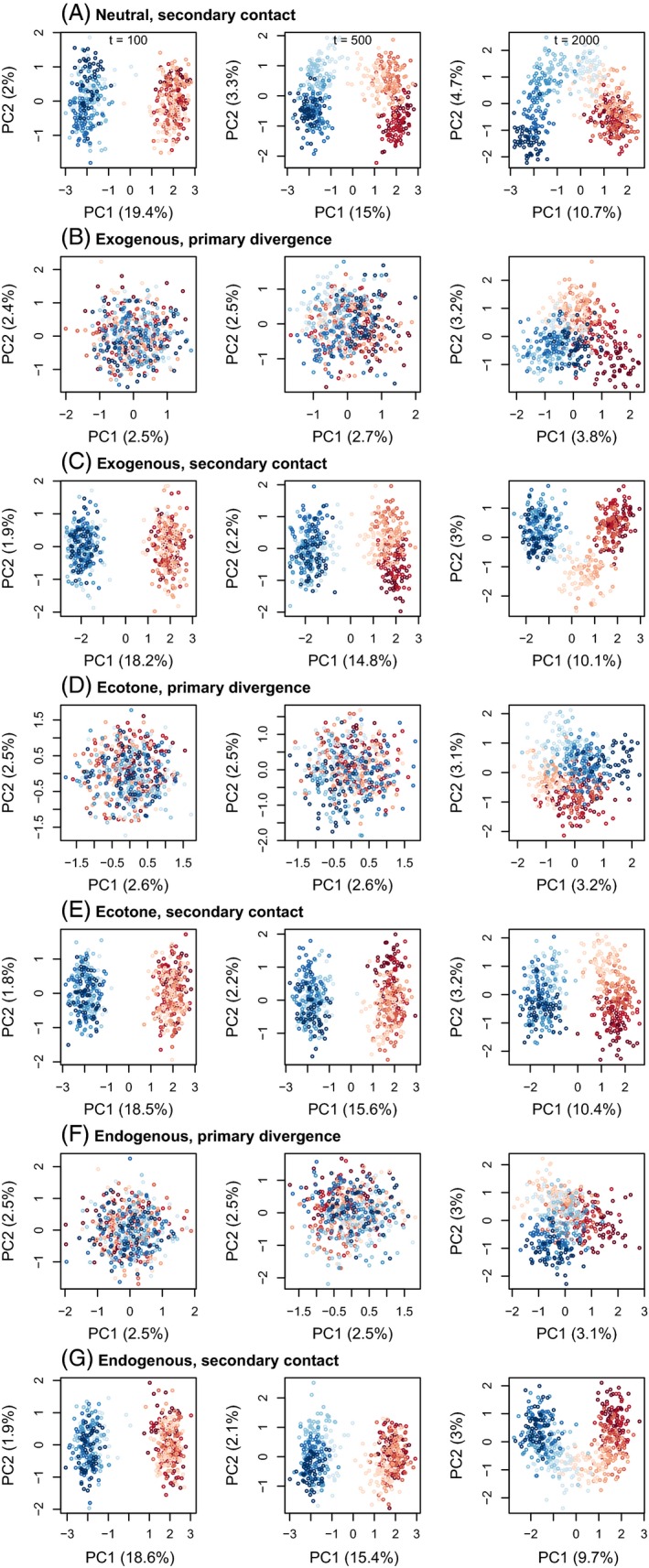
Scatterplots summarize patterns of genotypic variation for simulated data based on principal component analysis (PCA). Points denote individuals and are colored based on patch (dark red and dark blue for patches 1 and 10, with lighter shades indicating patches closer to the center). Results are shown for a migration rate of 0.001 and 100, 500, or 2000 generations. Results from a single simulation are shown, but replicate simulations produced qualitatively similar results. Clines from simulations with a higher migration rate of 0.01 are shown in Figure S3.

**Figure 3:**
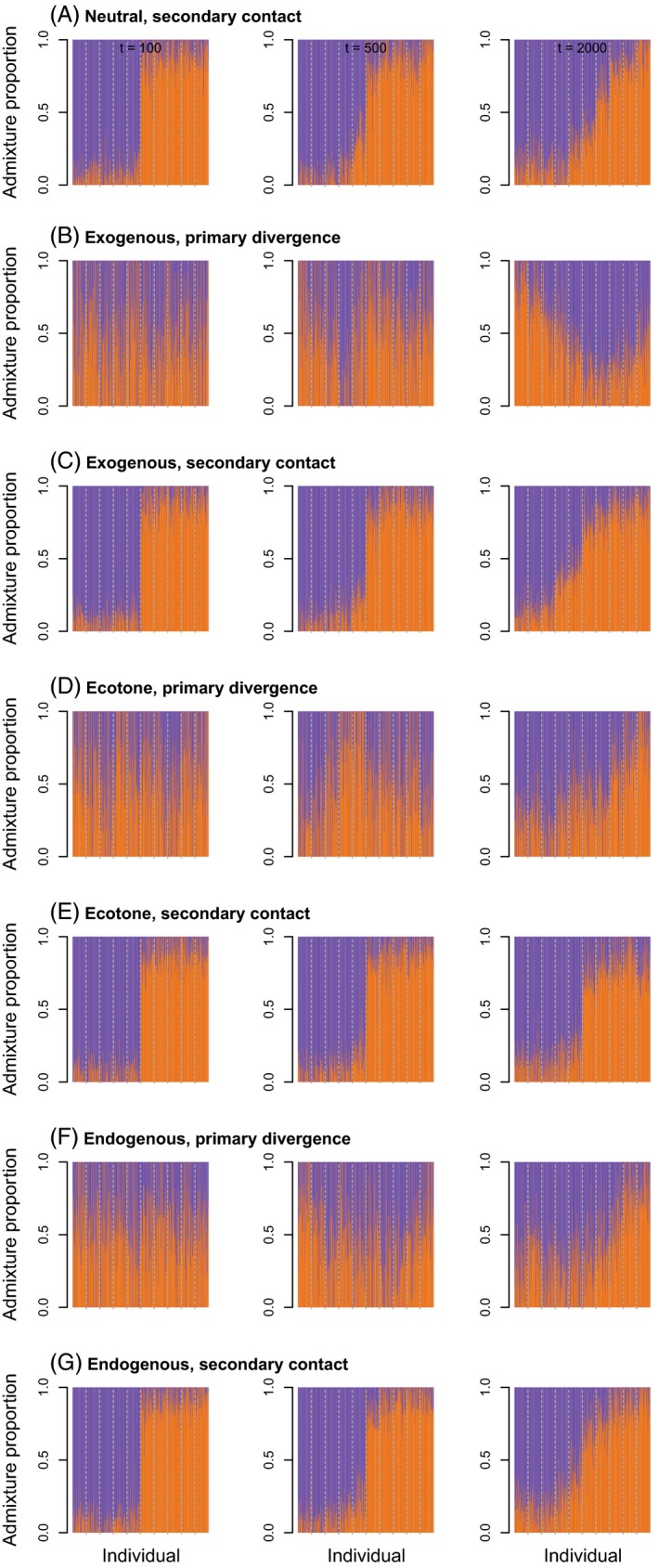
Barplots show maximum likelihood estimates of admixture proportions. Different colors denote ancestry from different hypothetical source populations. Here, we give results for a migration rate of 0.001 and 100, 500, or 2000 generations from a single set of simulations. Replicate simulations produced qualitatively similar results. Admixture from simulations with a higher migration rate of 0.01 is shown in Figure S4.

Our analyses show that time since the onset of secondary contact or primary divergence has a profound effect on patterns of genetic variation (Figs 1, 2, 3), even over the relatively short temporal scale of our simulations (2000 generations). By the end of the simulations, allele frequency clines were somewhat similar for both histories (i.e. secondary contact and primary divergence), despite clear differences earlier on. PCA and admixture proportions gave similar results. Thus, there may be a relatively narrow window of time during which can distinguish between these histories based on patterns of genetic or phenotypic data. With that said, time here is measured in generations, which could represent vastly different amounts of absolute time for species with different life histories and reproductive strategies (e.g. annual plants versus long‐lived, clonal trees).

At the end of the simulations (2000 generations), allele frequency clines and population structure were weak overall, particularly when the migration rate was 0.01 (Figures S2–S4). Phenotypic clines were much more pronounced and followed the environmental gradient or ecotone when exogenous selection occurred. This contrast is not surprising even though the neutral SNPs and quantitative trait SNPs were linked on a single chromosome, because without greater allele frequency differences among populations, limited linkage disequilibrium (LD) is expected. A lower migration rate slowed the decay of differences following secondary contact, but also resulted in smaller‐scale isolation by distance, including sharp phenotypic clines under the neutral secondary contact model, which could be incorrectly attributed to selection. Consistent with previous studies focused on equilibrium dynamics (Kruuk et al. 1999), we found that patterns of variation generated by exogenous and endogenous selection can also be difficult to distinguish earlier in the evolutionary process.

**Figure 4:**
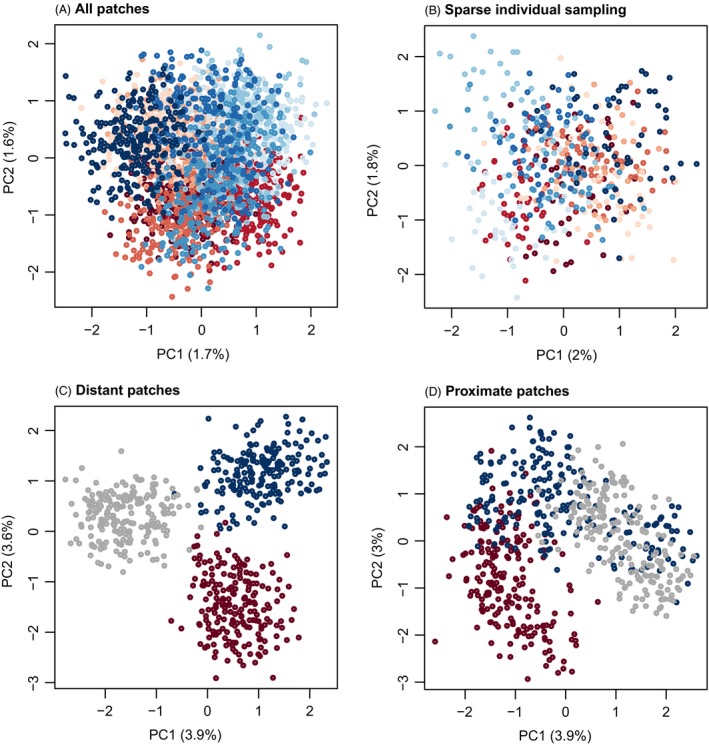
Principal component analysis (PCA) plots illustrate the effect of subsampling on summaries of genetic variation. Points denote individuals and are colored based on patch. Dark red, dark blue, and gray are used to denote peripheral and central patches when a subset of patches were sampled; otherwise dark red and blue indicate patches on opposite ends, with lighter colors used for more central patches. In panes (A) and (B), 50 or 5 individuals were included from each patch. In pane (C), 50 individuals were included from patches 1–4, 24–27 and 47–50, and in pane (D), 50 individuals were sampled from patches 20–31. Results are shown for a migration rate of 0.001 and 100, 500, or 2000 generations.

Neutral simulations that included 50 patches resulted in weak population structure overall, and this pattern was robust to sampling a smaller number of individuals per patch (5 vs 50; Fig. 4). However, other sampling approaches resulted in greater distortions of the true population structure. Sampling only center and edge patches resulted in three distinct genotypic clusters, which could be incorrectly interpreted as evidence of an isolated hybrid lineage or even hybrid species (e.g. Gompert et al. 2014). Even sampling only the central patches exaggerates levels of population structure. Together these results highlight the importance of broad geographic sampling to accurately recover clinal variation (also see Witherspoon et al. 2006; Schwartz and McKelvey 2009), as opposed to more limited sampling of putative hybrids and isolated ‘pure’ parental populations.

### Recommendations

Our illustrative simulations are consistent with other theoretical work on hybridization (e.g. Barton and Hewitt 1985; Kruuk et al. 1999; Barton and de Vladar 2009) and show that it will often be difficult to discriminate among different histories of selection and gene flow from genetic data. However, we show that even though primary divergence and secondary contact are thought of as hybridization and result in similar long‐term or equilibrium patterns of genetic variation (Barton and Hewitt 1985), recent primary divergence and secondary contact generate different patterns of variation. These differences occur because time is required for LD to buildup between neutral and selected variants with primary divergence (Barton and de Vladar 2009; Flaxman et al. 2014), whereas allele frequency differences between geographically isolated populations will generate LD upon secondary contact. This also means that, during the early stages of hybridization, secondary contact might often lead to segregation of greater functional (and nonfunctional) variation than primary divergence. On the other hand, early stages of primary divergence might be limited to sharp phenotypic and genetic differences for strongly selected characters (e.g. Poelstra et al. 2014; Soria‐Carrasco et al. 2014), with less segregating variation for other traits or genes in hybrids. We thus recommend that conservation and management practitioners treat recent primary divergence and secondary contact distinctly, as these processes can be distinguished and have different consequences. Once hybridization has occurred for a greater amount of time, patterns will become similar, and additional data, such as the phylogenetic relationship between or geographic distribution of hybridizing species, will be needed to parse these histories. Our results also show that widespread geographic sampling is important to accurately describe population structure and patterns of hybridization. As argued by practitioners of landscape genetics (e.g. Schwartz and McKelvey 2009), this means that structured, sensible sampling is preferable to sparse opportunistic sampling, or sampling focused on ends of a continuum.

Additional information will likely be gained from studies of hybridization that parse different types of genetic variants rather than treating them all in a single analysis (e.g. Gompert et al. 2014). For example, our simulations and discussion have considered genetic polymorphism, but we have focused on common rather than rare genetic variants. Rare variants, that is genetic variants with minor allele frequencies <1%, have become more accessible with current sequencing methods and could further help discriminate among different histories and provide information about recent evolutionary dynamics (Gravel et al. 2011; Mathieson and McVean 2012; Nelson et al. 2012). In particular, rare variants are often spatially restricted and can be informative about the dispersal of individuals from neighboring populations (Slatkin 1985; Barton and Bengtsson 1986; Gompert et al. 2014) and thus might provide better measures of contemporary gene flow among plant populations of conservation concern. Although more difficult to identify, genetic variants affecting important phenotypes or those linked to such variants could provide additional information if they are strongly structured by the environment (e.g. contrast phenotypic and neutral clines in Fig. 1). When one or a few genes of large effect determine functional phenotypes, it might be useful to examine patterns of genetic variation at these loci. However, when phenotypic variation is due to many variants with smaller effects, statistical approaches that combine information across genetic loci will be more useful (Berg and Coop 2014). Complementary methods that attempt to identify genetic variants potentially affected by selection in hybrids could also be used (e.g. Payseur et al. 2004; Gompert and Buerkle 2009, 2011). Thus, studies of hybridization between crops and wild species or native and non‐native plants, as well as gene flow in plants with fragmented populations, would benefit from an increased emphasis on the spread of functional genetic variation via hybridization (e.g. Rieseberg and Willis 2007; Hufford et al. 2013). Such information is needed to determine the fitness consequences of hybridization and thus to decide when hybridization should be valued, allowed or prevented.

## What, if anything, are hybrids

As noted above, there is a long history of recognizing phenotypically intermediate individuals as putative hybrids between differentiated parental populations or species, including the use of multivariate phenotypic analysis (Alston and Turner 1962; Hatheway 1962; Freeman et al. 1991) and gaining understanding of evolutionary relationships through crossing studies (e.g. Heiser 1947, 1956; Rieseberg 2000). The advent of molecular markers gave rise to the use of genetic information as the basis of inference of ancestry and the recognition of hybrids (e.g. Harrison and Arnold 1982; Vanlerberghe et al. 1986; Barton and Gale 1993). A variety of statistical models exist to support the recognition of hybrids and their distinction from individuals from parental populations (including species), both using population genetic (Boecklen and Howard 1997; Barton 2000; Pritchard et al. 2000; Anderson and Thompson 2002; Falush et al. 2003) and tree‐based models (Durand et al. 2011; Patterson et al. 2012). These various models and their implementation in software allow quantitative, model‐based recognition of hybrids, given sufficient, informative genetic data (Anderson and Thompson 2002; Falush et al. 2003; Vaha and Primmer 2006). Yet, the papers that describe these models often include explicit cautionary statements regarding the difficulty of distinguishing among different hybrid genealogies, as well as distinguishing hybrids from parentals (e.g. Barton 2000; Anderson and Thompson 2002). Aside from the problem of alleles shared between parental taxa and the resulting imperfect information about ancestry from allelic state, hybrids can be difficult to recognize simply because genetic recombination and sexual reproduction in different genealogies can lead to the same, ambiguous combination of alleles in genotypes. While the genetic variation that results from hybridization is known, it is not clear that as biologists we appreciate the extent to which different hybrid genealogies can lead to the same genetic composition. To illustrate the overlapping expectations for ancestry and genotypic composition of hybrids, we present a simple set of simulations in this section (reprising related simulations and results in Fitzpatrick 2012; Gompert et al. 2014; Lindtke et al. 2014), and their continuous variation along multiple dimensions of hybridization. These illustrations lead to the conclusions that it can be misleading to think about ancestry categories of hybrids and that hybrids will often be genetically and functionally diverse.

The fractional contribution of two (or more) parental taxa to the ancestry of hybrids is a common measure of hybridity and ancestry and is typically referred to as a hybrid index (Barton and Gale 1993; Boecklen and Howard 1997; Buerkle 2005) or admixture proportion (Pritchard et al. 2000; Falush et al. 2003). In the simple case of putative hybridization between two parental taxa, the hybrid index or admixture proportion (*q*) corresponds to variation along a single axis, with parental ancestry at each end and hybrids intermediate. Summarizing admixture in this way is very common, but it also disregards important information about the history of admixture (Barton 2000; Anderson and Thompson 2002; Fitzpatrick 2012; Lindtke et al. 2012; Gompert et al. 2014; Lindtke et al. 2014). For example, $F_{1}$ individuals will have a hybrid index of 0.5, but this is also the expected (mean) hybrid index of any $F_{2}\cdots F_{n}$ hybrid individuals, which do not have one of the parental taxa as a parent after the first generation of hybridization (i.e. they have experienced no backcrossing). Consequently, whereas a hybrid index does quantify a continuum of genetic hybridity and is preferable to a categorical analysis, it cannot discriminate among very different genealogies, including the differences in ancestry between an $F_{2}$ and an $F_{20}$. Additional information can be obtained from a second dimension of admixture, the fraction of loci that combine ancestry from the two parental taxa, which has been referred to as interspecific heterozygosity or interpopulation ancestry (denoted $Q_{12}$ here; Barton 2000; Fitzpatrick and Shaffer 2007; Fitzpatrick 2012; Lindtke et al. 2012; Gompert et al. 2014; Lindtke et al. 2014). Some software models this parameter explicitly from genetic data in hybrids and source populations (e.g. HIest and entropy; models for interpopulation ancestry are described in Fitzpatrick 2012; Gompert et al. 2014), but the most commonly used software for admixture analysis does not (structure; Pritchard et al. 2000; Falush et al. 2003). The combination of admixture proportion (*q*) and interpopulation ancestry ($Q_{12}$) contains additional information about admixture histories and thus is a general tool for summarizing the genomic composition of hybrids. For one, it allows identification of individuals that had a parental taxon as an immediate parent (including $F_{1}$ and any backcrossed hybrids), as these have maximal $Q_{12}$ for a given *q*.

### Simulations and analyses

As has been done in previous studies (Fitzpatrick 2012; Gompert et al. 2014; Lindtke et al. 2014), we performed individual‐based simulations of hybridization. In the first set, we repeatedly modeled two generations of hybridization that included parental, $F_{1}$, $F_{2}$, and backcross (BC) individuals. In a second set, we used replicates to generate expectations for the ancestry of $F_{2}$, $F_{5}$ and $F_{20}$ individuals. The simulations were of finite populations of 50 individuals that contribute to the parentage of any set of progeny ($F_{1}$, $F_{2}$, etc.). Diploid meiotic recombination and segregation were modeled, with 1000 marker loci distributed across 10 chromosomes, and a single, randomly located crossover per chromosome in each gamete. Thus, we were able to track ancestry with complete knowledge. To superimpose allelic states (including shared alleles between parental taxa and polymorphism within), we utilized an *F*‐model for shared ancestry of parental taxa and the genetic drift they experienced relative to the common ancestor (as above, and in Balding and Nichols 1995; Falush et al. 2003), with a beta distribution of allele frequencies in the ancestral population with parameters *α* and *β* equal to 0.8 (this distribution has a mean allele frequency of 0.5 and a standard deviation of 0.31). We set $F_{\mathrm{ST}}=0.5$ and only considered a random subset of 1000 marker loci with a minor allele frequency >0.05 in the sampled individuals (i.e. what are typically referred to as ‘polymorphic’ loci or common variants). We arbitrarily sampled 20 individuals of each of the parental taxa, 20 $F_{2}$, and 10 each $F_{5}$, $F_{20}$, and BC to each parental taxon. The simulations were performed in R (version 3.2.2; R Development Core Team 2015) and the script to perform the simulations is in the Supporting information.

**Figure 5:**
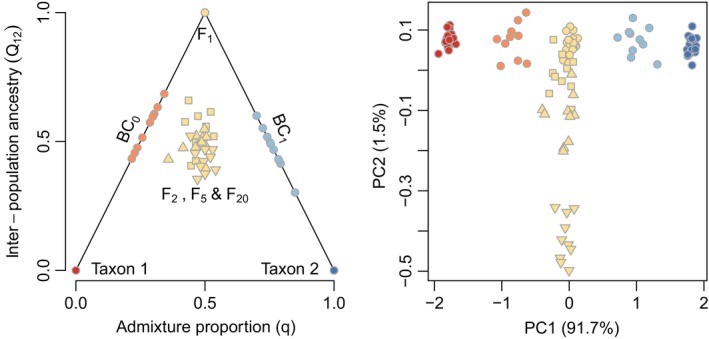
Ancestry for simulated individuals from parental taxa (Taxon 1 and 2) and hybrids vary in admixture proportion (*q*) and the fraction of loci at which individuals have ancestry from both parental taxa ($Q_{12}$, interpopulation ancestry; left pane of plot). Hybrids that are progeny from a cross involving one (BC) or both ($F_{1}$) parental taxa have maximal interpopulation ancestry for a given admixture proportion (on the edges of the triangle). In contrast, progeny from crosses between hybrid individuals ($F_{2}\cdots F_{n}$) has less than maximal interpopulation ancestry for a given admixture proportion. Principal component analysis (PCA) of genetic covariances among individuals in the simulated population (right pane) shows that genetic differences between the parental species (ancestry variation) constitute the dominant axis of genetic variation (colors as in left pane). $F_{1}\cdots F_{n}$ are genetically intermediate on PC1, and across all hybrids, PC1 mirrors the admixture proportion. $F_{20}$ individuals (downward‐pointing triangles) are distinguishable genetically from earlier $F_{n}$ hybrids and in general PC2 is associated with genetic variation among $F_{n}$ generations.

Our simulations tracked both the ancestry and allelic state of loci, and we present summaries of both (Fig. 5). Because we simulated admixture, we had perfect knowledge of the admixture proportions and interpopulation ancestry rather than needing to infer them. If one were to infer ancestries based on models and software (e.g. Gompert et al. 2014), there would be more uncertainty and variance around the true values shown here (uncertainty in ancestry is inversely proportional to allele frequency differences between the parental taxa, that is, to the extent that allelic state is informative about ancestry). With the level of allele frequency difference between our parental populations ($F_{\mathrm{ST}}=0.5$), recognition of parental individuals and distinguishing them from all hybrids was unambiguous with PCA (Fig. 5, PCA performed in R; R Development Core Team 2015; it would be more difficult to distinguish parental and hybrid individuals based on allelic state if parental populations were more similar genetically). In terms of ancestry, $F_{1}$ individuals were distinguishable from more advanced generation $F_{n}$ hybrids ($F_{2}$, $F_{5}$, and $F_{20}$) and backcrossed individuals on the basis of their maximal interpopulation ancestry. Likewise, BC individuals are recognizable on the basis of their maximal interpopulation ancestry for a given admixture proportion. Distinguishing among different generations of backcrossing (e.g. whether $F_{1}$, or $F_{2}$ was hybrid parent) would not be possible based only on the information contained in $Q_{12}$ and *q* (knowledge of chromosomal blocks of ancestry would be helpful; Gompert and Buerkle 2013). Segregation even in the $F_{1}$ is highly variable and ancestry in later generation hybrid $F_{n}$ parents is expected to overlap with that of the $F_{1}$. More generally and as noted in previous research, discriminating between genealogies beyond the first two generations of admixture is difficult (Barton 2000; Anderson and Thompson 2002) without additional information. This is illustrated in these simulations by the overlapping expectations for $Q_{12}$ and *q* across the individuals in the $F_{2}$, $F_{5}$, and $F_{20}$ generations. While drift would cause $Q_{12}$ to decline over further generations and ultimately lead to the fixation of ancestry states in finite populations over time (Stam 1980; Chapman and Thompson 2002, 2003; MacLeod et al. 2005; Buerkle and Rieseberg 2008), 20 generations are insufficient to have a detectable effect in a simulated population of 50 individuals.

If hybridization is restricted to two generations, with sufficient genomic sampling, it can be possible to identify different parental, $F_{1}$, $F_{2}$, and BC categories. Here, we have considered perfect knowledge of ancestry and previous work has addressed sources of uncertainty that would lessen the prospects for clear expectations for ancestry (Anderson and Thompson 2002; Vaha and Primmer 2006; Burgarella et al. 2009; Fitzpatrick 2012). But beyond the issues of whether hybridization involves only two generations of hybridization and uncertainty in empirical ancestry estimates, analysis of ancestry categories is problematic because these classes mask the fact that they will contain genetic and functional phenotypic variation. Perhaps we stand to be the most misled in the case of $F_{1}$ individuals, where allelic polymorphisms in the parental populations will result in genetically variable $F_{1}$ individuals, contrary to the expectation for a single genotype of $F_{1}$ resulting from typical experimental crosses between homozygous parents. This genetic variation is evident among $F_{1}$ individuals in our simulations (Fig. 5). This variation would be even greater if one considered $F_{1}$ individuals from different geographic locations, where allele frequencies in parental taxa are likely to differ even more (e.g. Gompert et al. 2014; Mandeville et al. 2015). Somewhat similarly, categorical treatment of ancestry in hybrids leads to overlapping expectations for $Q_{12}$ and *q* in $F_{2}$, $F_{5}$, and $F_{20}$ individuals, but analysis of their allelic states shows that $F_{20}$ individuals differ genetically from early $F_{n}$ generations (PCA in Fig. 5). These genetic differences could be responsible for functional phenotypic differences and comparable functional differences might arise from more subtle genotypic differences between $F_{n}$ generations.

Overall, the use of the term *hybrid classes* or *categories*, and methods for their inference, could obscure important variation that exists within classes. Instead, Rieseberg and Carney (1998) suggested it is worthwhile to focus on the fitness of individual genotypes, rather than hybrid classes. Certainly, our simple model illustrates that it can be nonsensical to refer to the ‘fitness of hybrids’, as the genetic and ancestry composition of hybrids can be highly variable and hybrids would be expected to vary substantially for phenotypes. Given the expected genetic and phenotypic variability within hybrids, and the potential for transgressive phenotypes (Rieseberg et al. 1999a, 2003b), discussion of hybrid fitness should be in the context of the typical complexity of tying phenotype (including fitness) to genotype in natural populations, which is particularly difficult in variable environments and in variable genetic backgrounds (Weiss 2008; Rockman 2012).

### Recommendations

In both applied and basic science settings, knowledge of the existence and attributes of hybrids can provide a foundation for learning about species interactions and maintenance (Arnold 2006; Allendorf et al. 2013). For example, a predominance of BC hybrids would lead to genetic exchange between parental taxa and a potential local erosion of species differences, whereas if hybrids are restricted to relatively abundant $F_{n}$ individuals, these will affect the demography of parental taxa through wasted reproductive effort on $F_{1}$ hybrid progeny and possibly through competition. Our simple model reflects our understanding that the ancestry and genetic composition of hybrids vary along multiple axes and treatment of hybrids as a singular entity would disregard potentially important variation. Thus, management decisions might need to consider the types of hybrids generated and could even accommodate different actions for different hybrids within the same biological system.

Furthermore, hybrids beyond the $F_{1}$ will also vary in ancestry along their chromosomes, both in tracts of ancestry that have not yet recombined, and as a result of drift and selection leading individual loci to deviate from the average ancestry in the genome (reviewed in Gompert and Buerkle 2013). For this reason, hybrids have been of interest to evolutionary biologists who are interested in the genetics of species boundaries (Rieseberg et al. 1999b; Rieseberg and Buerkle 2002; Buerkle and Lexer 2008; Payseur 2010; Gompert et al. 2012). Incomplete reproductive isolation and hybridization have provided support for the ‘genic view’ of speciation and species boundaries (Wu 2001; Abbott et al. 2013). Additionally, recent studies in a variety of taxa have drawn attention to variability in the genetic outcomes of hybridization that followed secondary contact between the same pairs of species in multiple locations or contexts (Rieseberg 2006; Nolte et al. 2009; Teeter et al. 2010; Lepais and Gerber 2011; Lagache et al. 2013; Gompert et al. 2014; Mandeville et al. 2015). For both applied and basic evolutionary biology, this variability in outcomes means that it can be difficult to formulate categorical statements about the composition, importance, and likely conservation threats of hybrids. The empirical abundance of parental taxa and hybrids at one site may or may not be informative about other locations where the taxa co‐occur (e.g. Aldridge and Campbell 2009; Mandeville et al. 2015). Likewise, as noted above, genetic variation in parents and hybrids makes it difficult to make categorical statements about the genotypes, phenotypes, and fitness of hybrids (e.g. Sweigart et al. 2007; Good et al. 2008). This challenge is not a matter of uncertainty that arises from analytical approaches and software, but is inherent to the process of hybridization, as we have illustrated with the simulations in this paper.

Overall, these complexities mean it will be difficult to know the consequences of hybridization without detailed study (Allendorf et al. 2013), which could include estimation of multiple dimensions of ancestry (Gompert and Buerkle 2013), sampling multiple geographic locations and contexts (e.g. Hamilton et al. 2013; Haselhorst and Buerkle 2013; Gompert et al. 2014; Mandeville et al. 2015), and characterization of the demography of parental and hybrid individuals in populations (e.g. Carney et al. 2000; Fitzpatrick and Shaffer 2007).

## Synthesis and conclusions

A common theme of our results and discussion is that hybridization is not a singular process, but rather a collection of related processes with variable outcomes and consequences. In support of this, as noted above, empirical studies have often documented variation in outcomes of hybridization in different locations or contexts, in terms of the genomic composition of hybrids, patterns of introgression, and the ecological consequences of hybridization (e.g. Yanchukov et al. 2006; Lepais et al. 2009; Nolte and Tautz 2010; Teeter et al. 2010; Nice et al. 2013; Gompert et al. 2014; Mandeville et al. 2015). Indeed, consistent outcomes of hybridization appear to be mostly limited to taxa that exhibit limited intraspecific variation for loci affecting fitness and where endogenous selection dominates (e.g. Buerkle and Rieseberg 2001). Such variability limits our ability to predict the outcome of specific instances of hybridization and thus is relevant for our understanding of evolutionary biology in general and has practical consequences for management. For example, invasion by a non‐native species could result in extirpation of a native species in one area but not in another, or transgene escape from a crop could occur readily into some wild populations but not others. Thus, it might be difficult to make valid general statements about the threats or benefits of hybridization, even for individual species. Likewise, extrapolation from single empirical examples (i.e. studies or sites) could be problematic. Clearly, such problems will be exacerbated when species exhibit substantial isolation by distance or local adaptation and fail to function as cohesive entities. We conclude by noting that while we have focused on hybridization between pairs of diploid populations or species, the points we have made should also apply when hybridization generates polyploids or involves multiple species. However, in such cases, even more factors could affect the ecological and evolutionary dynamics, rendering the outcomes of these instances of hybridization even less predictable.

## Data archiving statement

Data available from the Dryad Digital Repository: http://dx.doi.org/10.5061/dryad.g7g45.

## Supporting information


**Figure S1.** Diagram summarizing the spatial layout and sampling of populations for the ‘What, if anything, is hybridization’ simulations.
**Figure S2.** Plots show neutral allele frequency (gray) and quantitative trait (orange) clines from simulated data with a migration rate of 0.01.
**Figure S3.** Scatterplots summarize patterns of genotypic variation for simulated data based on PCA.
**Figure S4.** Barplots show maximum likelihood estimates of admixture proportions.

**Data S1.** R code used to simulate and analyze hybrids.
