# Supplementary material for: What, if anything, are hybrids: enduring truths and challenges associated with population structure and gene flow
Source: Evol Appl. 2016 Apr 26;9(7):909–23. doi: 10.1111/eva.12380 (PMC4947152; doi:10.1111/eva.12380)
Supplement: Supplementary file 1 — Figure S1. Diagram summarizing the spatial layout and sampling of populations for the ‘What, if anything, is hybridization’ simulations. Figure S2. Plots show neutral allele frequency (gray) and quantitative trait (orange) clines from simulated data with a migration rate of 0.01. Figure S3. Scatterplots summarize patterns of genotypic variation for simulated data based on PCA. Figure S4. Barplots show maximum likelihood estimates of admixture proportions. [file EVA-9-909-s001.pdf]

Supplemental figures

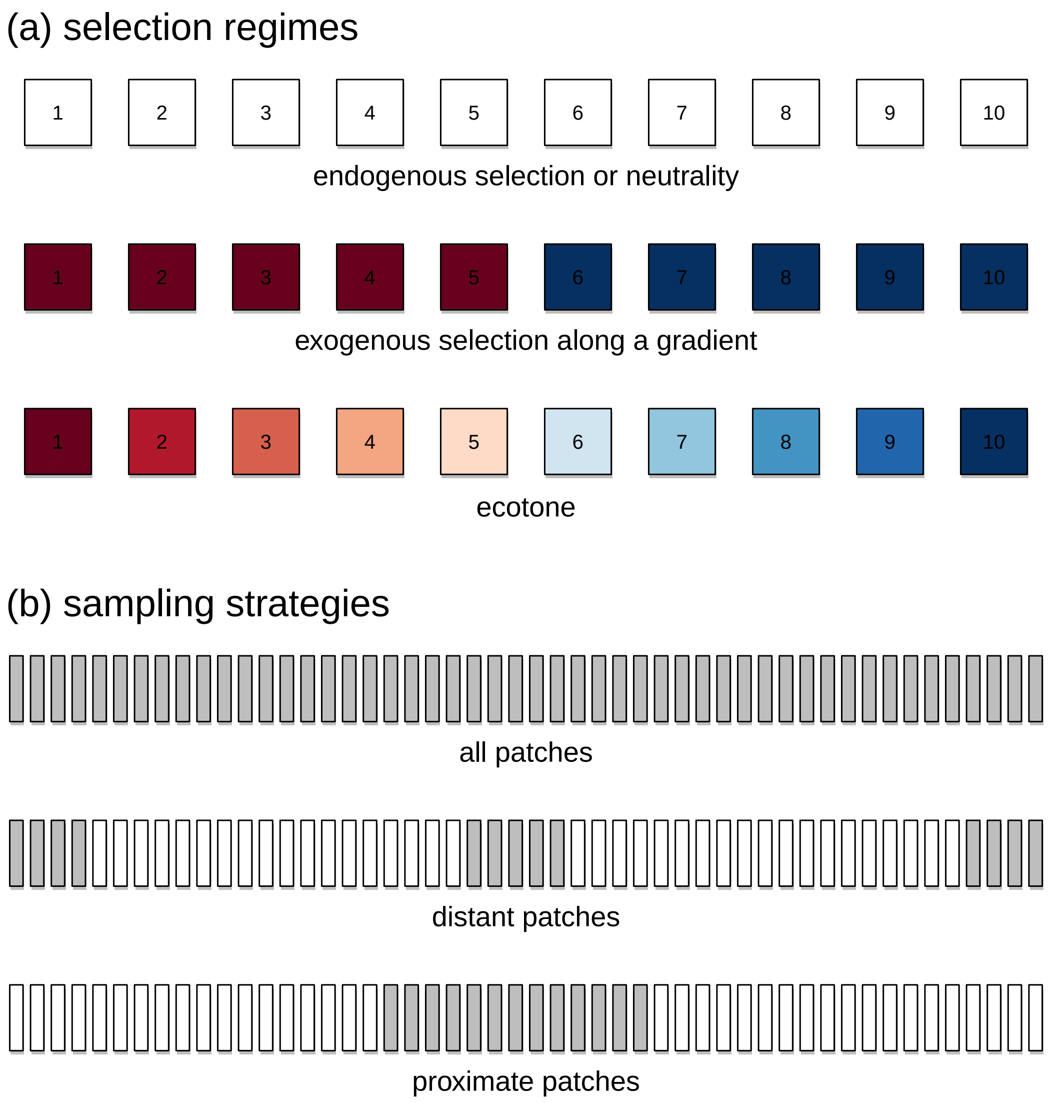

Figure S1: Diagram summarizing the spatial layout and sampling of populations for the 'What, if anything, is hybridization' simulations. (a) shows the 10 patches (numbered 1 to 10) for the main simulations. Rows denote different fitness schemes from different sets of simulations. White indicates no exogenous selection, dark red to light red boxes indicate optimal phenotypes of -0.5 to -0.1 (steps of 0.1 between patches), and light to dark blue boxes indicate optimal phenotypes of 0.1 to 0.5 (steps of 0.1). We assigned the same allele frequencies to all patches for the primary divergence simulations, but different allele frequencies to patches 1–5 and 6–10 for secondary contact. We allowed for migration between neighboring patches only. (b) shows the sub-sampling strategies we used for the 50 patch simulations. As described in the main text, we sampled different sets of patches, which are shown as the gray boxes in each row (white boxes denote un-sampled patches).

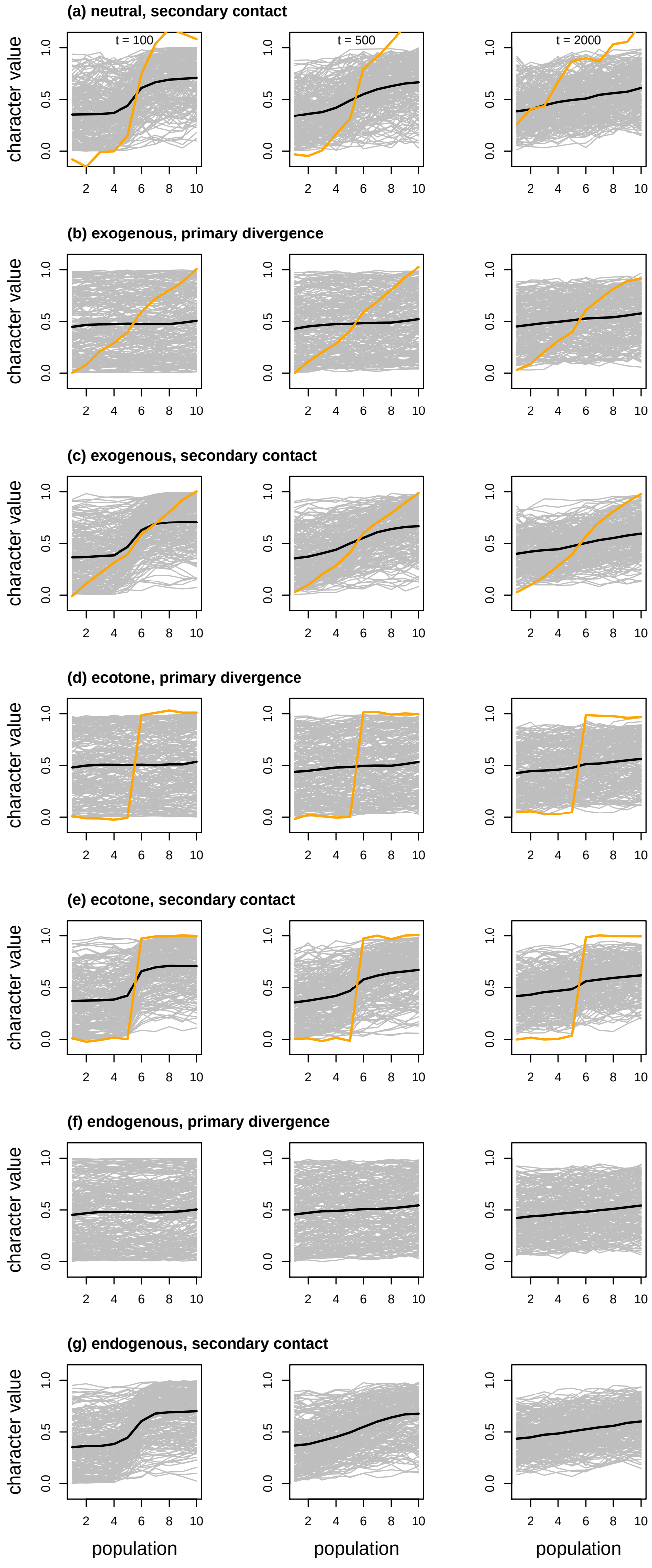

Figure S2: Plots show neutral allele frequency (gray) and quantitative trait (orange) clines from simulated data with a migration rate of 0.01. The mean allele frequency cline with SNPs polarized such that the allele plotted was rarer in patch 1 than patch 10 is depicted with a black line. Clines after 100, 500, and 2000 generations are shown. Results from a single simulation are shown, but replicate simulations produced qualitatively similar results.

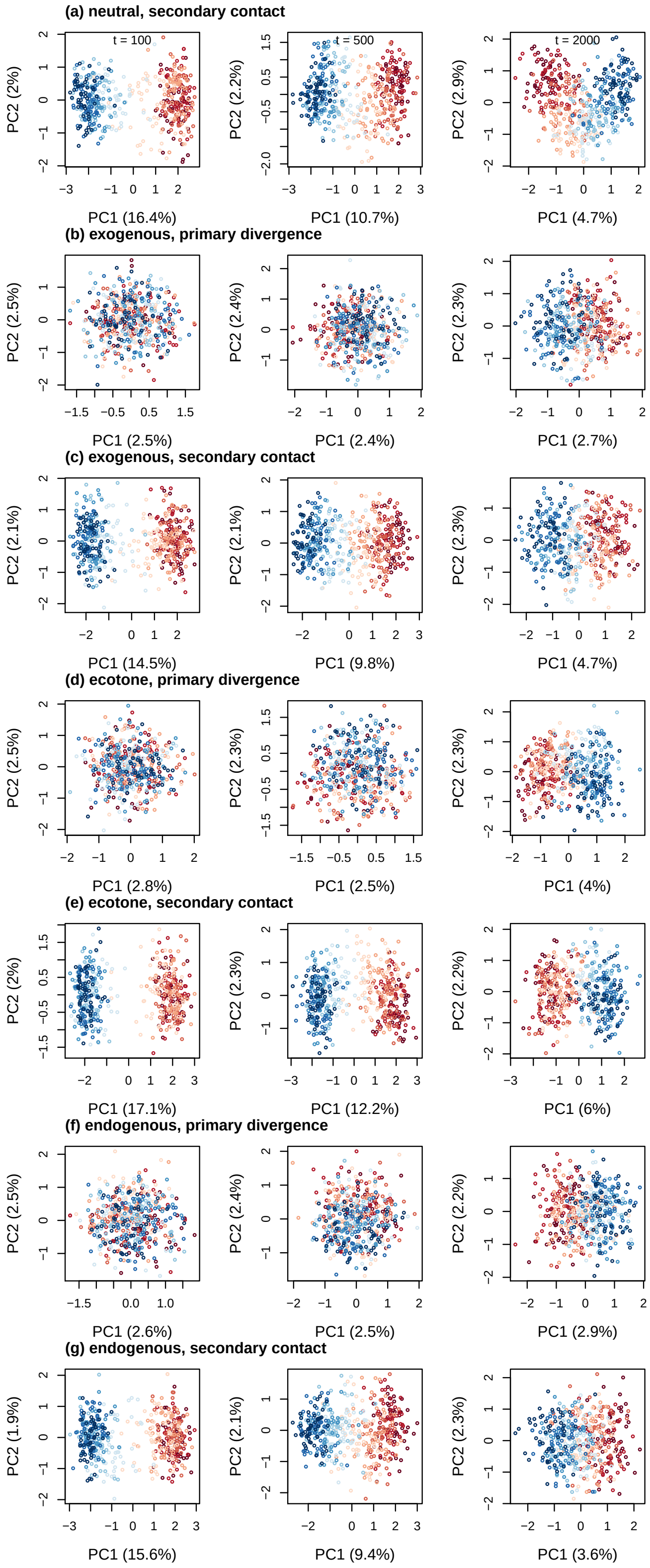

Figure S3: Scatterplots summarize patterns of genotypic variation for simulated data based on PCA. Points denote individuals and are colored based on patch (dark red and dark blue for patches 1 and 10, with lighter shades indicating patches closer to the center). Results are shown for a migration rate of 0.01 and 100, 500, or 2000 generations. Results from a single simulation are shown, but replicate simulations produced qualitatively similar results.

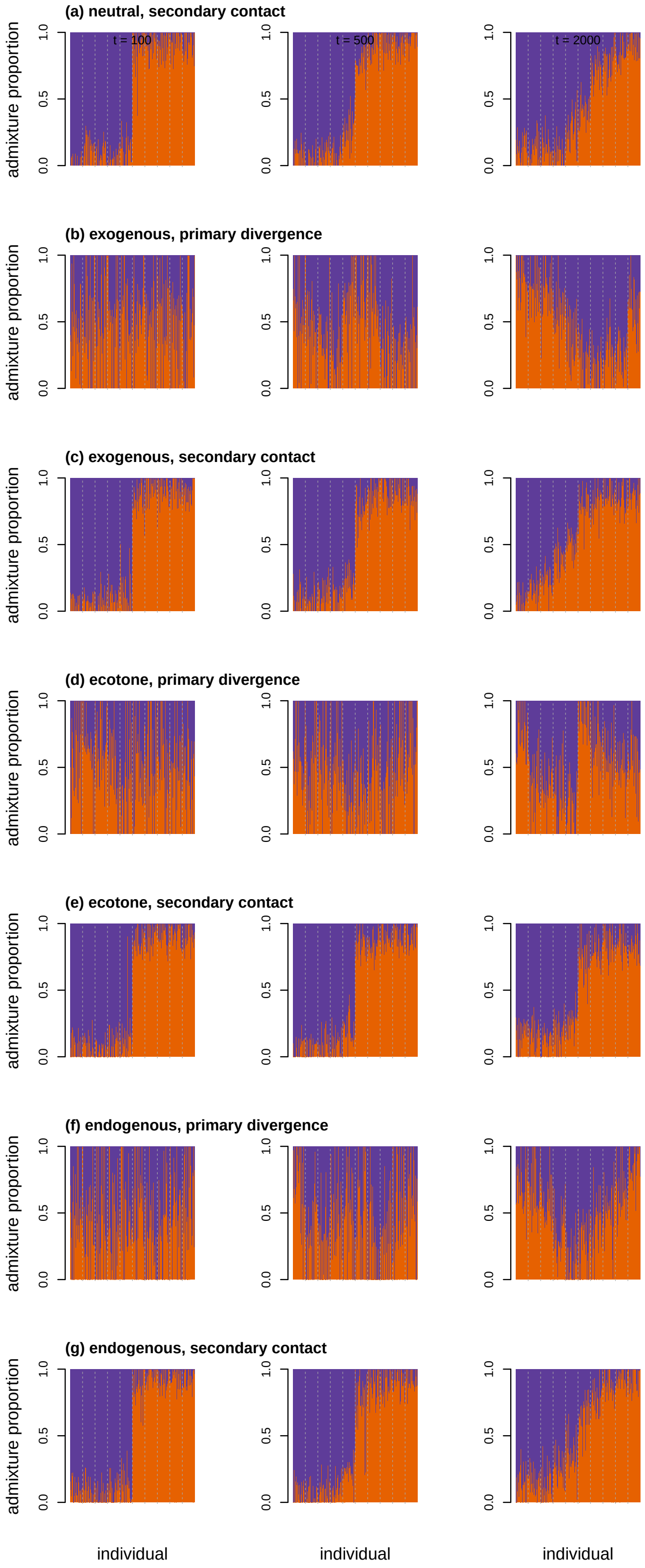

Figure S4: Barplots show maximum likelihood estimates of admixture proportions. Different colors denote ancestry from different hypothetical source populations. Here we give results for a migration rate of 0.01 and 100, 500, or 2000 generations from a single set of simulations. Replicate simulations produced qualitatively similar results.
